# Non-invasive assessment of portal hypertension by multi-parametric magnetic resonance imaging of the spleen: A proof of concept study

**DOI:** 10.1371/journal.pone.0221066

**Published:** 2019-08-20

**Authors:** Christina Levick, Jane Phillips-Hughes, Jane Collier, Rajarshi Banerjee, Jeremy F. Cobbold, Lai Mun Wang, Stefan K. Piechnik, Matthew D. Robson, Stefan Neubauer, Eleanor Barnes, Michael Pavlides

**Affiliations:** 1 Translational Gastroenterology Unit, University of Oxford, Oxford, England, United Kingdom; 2 Department of Radiology, Oxford University Hospitals NHS Trust, Oxford, England, United Kingdom; 3 Perspectum Diagnostics, Oxford, England, United Kingdom; 4 Department of Histopathology, Oxford University Hospitals NHS Trust, Oxford, England, United Kingdom; 5 Division of Cardiovascular Medicine, Radcliffe Department of Medicine, University of Oxford, Oxford, England, United Kingdom; 6 Oxford National Institute of Health Research Biomedical Research Centre, John Radcliffe Hospital, Oxford, England, United Kingdom; 7 University of Oxford, Oxford, England, United Kingdom; Medizinische Fakultat der RWTH Aachen, GERMANY

## Abstract

**Background and aims:**

Non-invasive assessment of portal hypertension is an area of unmet need. This proof of concept study aimed to evaluate the diagnostic accuracy of a multi-parametric magnetic resonance technique in the assessment of portal hypertension. Comparison to other non-invasive technologies was a secondary aim.

**Methods:**

T_1_ and T_2_* maps through the liver and spleen were acquired prior to trans-jugular liver biopsy and hepatic vein pressure gradient (HVPG) measurement. T_1_ measurements reflect changes in tissue water content, but this relationship is confounded by the presence of iron, which in turn can be quantified accurately from T_2_* maps. Data were analysed using LiverMultiScan (Perspectum Diagnostics, Oxford, UK) which applies an algorithm to remove the confounding effect of iron, yielding the “iron corrected T_1_” (cT_1_). Sensitivity, specificity, diagnostic values and area under the curve were derived for spleen cT_1_, liver cT_1_, transient elastography, and serum fibrosis scores. HVPG was the reference standard.

**Results:**

Nineteen patients (15 men) with median age 57 years were included. Liver disease aetiologies included non-alcoholic fatty liver disease (n = 9; 47%) and viral hepatitis (n = 4; 21%). There was strong correlation between spleen cT_1_ and HVPG (r = 0.69; p = 0.001). Other non-invasive biomarkers did not correlate with HVPG. Spleen cT_1_ had excellent diagnostic accuracy for portal hypertension (HVPG >5 mmHg) and clinically significant portal hypertension (HVPG ≥10 mmHg) with an area under the receiver operating characteristic curve of 0.92 for both.

**Conclusion:**

Spleen cT_1_ is a promising biomarker of portal pressure that outperforms other non-invasive scores and should be explored further.

## Introduction

Portal hypertension is the major contributor to liver disease complications including bleeding from oesophageal varices, ascites and hepatic encephalopathy. Direct portal pressure measurement is difficult as there is no direct connection between the portal and systemic circulations. In practice, the best surrogate for the true portal pressure is the hepatic vein pressure gradient (HVPG), which is considered the reference standard[1]. HVPG can risk stratify patients for the development of clinical outcomes[2–5], and is a Food and Drug Association (FDA) accepted surrogate end-point[6].

Portal hypertension is defined as portal venous pressure >5 mmHg. Clinically significant portal hypertension is defined as HVPG ≥10 mmHg and this incurs a risk of developing complications[4]. A HVPG ≥12 mmHg confers increased risk of oesophageal variceal bleeding[7]. HVPG measurement is also valuable in monitoring treatment effectiveness with non-selective beta-blockers[7]. Despite such important prognostic information being provided by HVPG, it is rarely measured in clinical practice, due to its invasiveness, cost and expertise required[8]. Non-invasive markers of portal pressure are therefore needed.

One of the most studied markers of portal hypertension is liver stiffness measured by transient elastography. This had an area under the receiving operator curve (AUROC) of 0.93 with 90% sensitivity and 79% specificity for diagnosing clinically significant portal hypertension in a meta-analysis of 18 studies[9], but its correlation with HVPG deteriorates above 12 mmHg[10]. Transient elastography has been recommended to risk stratify for clinically significant portal hypertension when HVPG is not available[11]. Transient elastography, however, is associated with high failure rates in overweight/obese patients[12] and patients with ascites, commonly observed in patients with portal hypertension. Other candidate markers to show associations with HVPG include blood-based parameters[13, 14] and spleen stiffness[15–17], but no non-invasive marker of portal hypertension has yet been supported by sufficient evidence to replace HVPG[11].

We have developed a non-invasive multi-parametric magnetic resonance (MR) technique that can be used to measure the “iron corrected T_1_ relaxation time” (cT_1_)[18]. When applied to the liver, this technology had excellent diagnostic accuracy for the assessment of liver fibrosis and inflammation compared with biopsy[19, 20] and could predict the development of clinical outcomes[21].

Portal hypertension results in spleen congestion and increased splenic water content, and if chronic, may cause splenic fibrosis[22, 23]. We hypothesised that these pathophysiological changes would result in increased spleen cT_1_ which can be measured using our multi-parametric MR technique.

This proof of concept study aimed to evaluate the diagnostic accuracy of spleen cT_1_ as a biomarker of portal hypertension using HVPG as the reference standard. Liver cT_1,_ liver transient elastography and serum based biomarkers were also evaluated.

## Patients and methods

### Study design and patient population

This was a prospective, proof of concept study. Patients were recruited consecutively from the John Radcliffe Hospital (Oxford, UK) between August 2013 and April 2015. Eligible patients were aged 16 years or over who were referred for liver biopsy for suspected cirrhosis with/without portal pressure measurement. All patients consented to portal pressure measurement, so clinical referrals for percutaneous liver biopsy were changed to trans-jugular biopsy after the patient’s informed consent. Patients were excluded if they had contraindications to MR scanning, or if the HVPG could not be computed from the available pressure measurements. See S1 Text for more details about the recruitment strategy.

Patients attended a single study visit for MR scanning and liver transient elastography using Fibroscan (Echosens, France). Participants attended having fasted for ≥4 hours and could take their usual medications. Blood was drawn for laboratory tests to compute serum based indices of liver fibrosis (AST/ALT ratio[24], APRI[25], FIB-4[26]). MR scans and other non-invasive scores were analysed blinded from the liver biopsy and HVPG results.

The study was conducted according to the principles of the 1975 Declaration of Helsinki and ethical approval was granted from the UK National Research Ethics Service (13/SC/0243). All patients gave written informed consent.

### Transient elastography

TE was performed using Fibroscan (Echosens, France), with the patient lying supine and with the right arm fully extended. The medium (M) probe was used first and if unsuccessful or unreliable results were obtained then the extra-large (XL) probe was tried. Ten measurements per patient were needed for a successful scan and the manufacturer’s recommendations were used to assess the validity of each examination (10 measurements in each patient, with a rate of successful measurements greater that 60% and where the interquartile range was not greater than 0.3 of the median).

### HVPG measurement and trans-jugular liver biopsy

The procedures were performed under fluoroscopic guidance by experienced interventional radiologists, who were blinded to the MR and other non-invasive assessments results. Participants attended after an overnight fast and had the procedures performed under local anaesthetic and conscious sedation. Portal pressure measurements were done according to local routine practice using a straight tipped catheter connected to a digital pressure recording device (Datex Ohmeda S/5, GE Healthcare, Kentucky, USA) through a pressure transducer (Namic, Navilyst Medical, Massachusetts, USA). Pressure readings were allowed to stabilise for 30–40 seconds before being recorded. Free hepatic vein pressures (FHVP) were measured 2–4 cm from the confluence of the hepatic vein with the inferior vena cava, and wedged hepatic vein pressures (WHVG) were obtained by advancing the catheter until a wedged position was achieved. HVPG was calculated as the difference between these measures (HVPG = WHVP–FHVP). Liver biopsies were taken using a 19G trans-jugular biopsy needle after the pressure measurements.

### Liver biopsy reporting

Liver biopsies were analysed for Ishak fibrosis stage (0–6) according to the local clinical routine. This involves consensus reporting by two experienced pathologists and discussion in a clinico-pathological meeting before the final report is issued. The pathologists were blinded to the MR and HVPG results. Ishak stage ≥F5 defined cirrhosis. The aetiology of liver disease was defined according to standard clinical criteria using histology, clinical information and laboratory results.

### MR data acquisition

The multi-parametric MR protocol and its application to measure liver cT_1_ has been previously described[19, 21]. The same methodology was used here to acquire T_1_ and T_2_* maps in the same axial upper abdominal slices through the liver and spleen. The acquisition of T_1_ and T_2_* maps can be achieved in as little as three 10-second breath holds (one breath hold to acquire localiser images, and one each for the T_1_ and T_2_* maps). Proton MR spectroscopy was performed for liver fat quantification, acquired in 15 minutes. All scans were performed on a 3-Tesla MRI scanner (Tim Trio, Siemen’s Healthcare, Germany).

### T_1_ correction for iron

Changes in tissue free water content lead to respective changes in T_1_ relaxation time, which can be measured accurately using MR. Tissue iron accumulation, however, has an opposing effect. Our method, which was developed specifically for the liver, applies a correction algorithm to remove this confounding effect, yielding the iron corrected T_1_ (cT_1_). In this algorithm, iron is quantified using T_2_* maps acquired through the same transverse slice as the T_1_ maps. The algorithm was applied verbatim to the spleen in this work.

### MR data analysis

**Fig 1:**
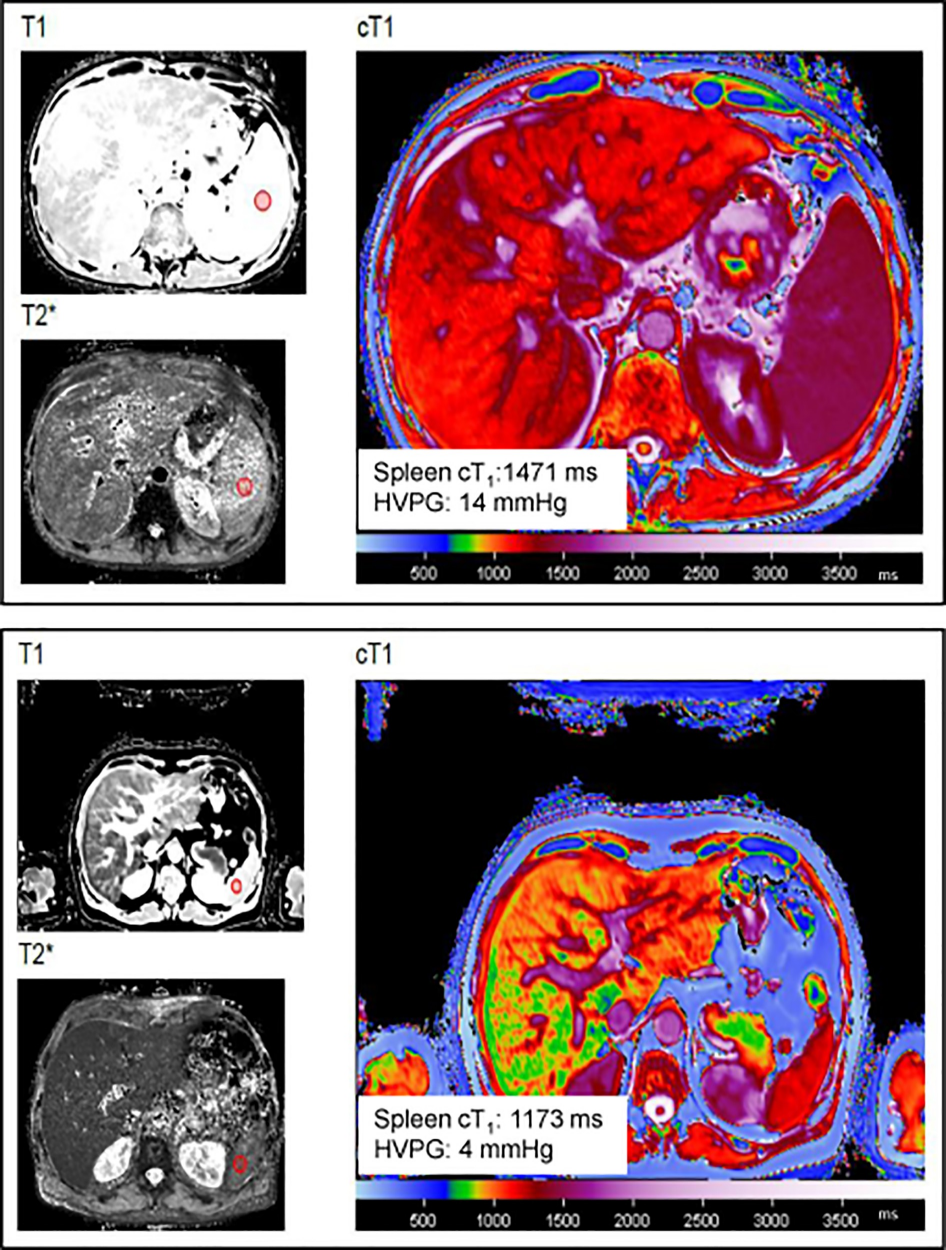
Measurement of spleen cT_1_ using LiverMultiScan. Representative images from a patient with (top panel) and without (bottom panel) portal hypertension. Red circles on the T_1_ and T_2_* maps represent the region of interest where the measurements were taken. Abbreviations: HVPG: Hepatic vein pressure gradient, cT_1_, iron corrected T_1_.

LiverMultiScan software (Perspectum Diagnostics, Oxford, UK) was used to analyse the MR data. The software uses T_1_ and T_2_* data to produce a quantitative cT_1_ map. Regions of interest (ROI) ≥1cm^2^ were drawn within the liver and spleen away from vessels, biliary structures and organ boundaries to determine mean cT_1_ values. Fig 1 demonstrates image analysis for spleen cT_1_. ROIs were acceptable only if R^2^ (co-efficient of determination) was ≥ 97% in both the T_1_ and T_2_* maps. For liver data, two ROIs were drawn, one in each lobe and the mean of the two used in the analysis. The MR analysis was performed blinded from liver biopsy and HVPG results. MR spectroscopy data were analysed using AMARES in the jMRUI package. Liver fat content was expressed as the proportion of the total signal.

### Statistical analysis

The primary variable of interest was spleen cT_1_. Liver cT_1_, liver stiffness and simple serum based fibrosis panels (AST/ALT, APRI, FIB-4) were secondary variables of interest. HVPG was the primary reference standard. Full data sets were available for the primary variable of interest and reference standard. Missing data for variables of secondary interest were handled by pairwise deletion (available case analysis). Spleen and liver cT_1_ were tested for the diagnosis of (a) any degree of portal hypertension (HVPG >5 mmHg) and (b) clinically significant portal hypertension (HVPG ≥10 mmHg). A comparison was carried out against liver histology for the diagnosis of liver cirrhosis.

Descriptive statistics were used to summarise subject characteristics. The Mann-Whitney and chi-squared tests were used for differences between patients with and without clinically significant portal hypertension or cirrhosis. Normality was tested using Shapiro-Wilk analysis. Associations between continuous variables were tested using the Pearson’s correlation coefficient for parametric variables and Spearman’s rank correlation coefficient for non-parametric variables. Receiver operating curve analysis was used for the assessment of diagnostic accuracy. Cut-off values to maximise sensitivity and specificity were recorded. Univariate linear and logistic regression analyses were carried out for prediction of HVPG and CSPH respectively. Non-invasive parameters with p<0.1 were added to multivariate models. The level of statistical significance was set at p>0.05. Statistical analyses were performed using Graph Pad Prism (v6.04) and SPSS (v22, Armonk NY, IBM Corp).

## Results

**Fig 2:**
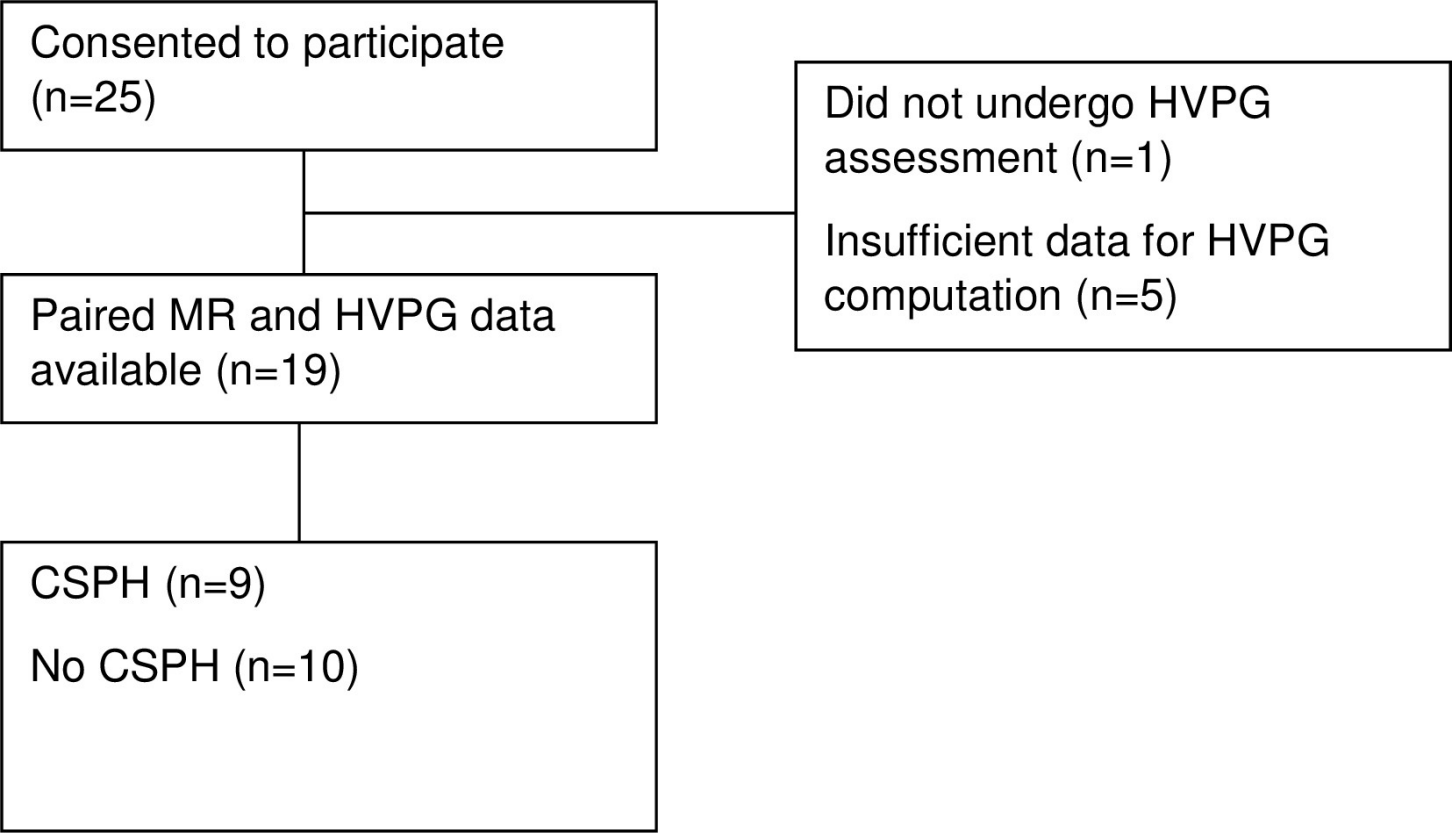
Study flow diagram. Abbreviations: **MR:** magnetic resonance, **HVPG:** hepatic vein pressure gradient, **CSPH:** clinically significant portal hypertension.

Twenty-five patients were recruited and nineteen were included in the final analysis. The median (IQR) interval between the study visit and the HVPG measurement was 5 days (1–15). Six participants were excluded from analysis due to incomplete study assessments (Fig 2). Of the excluded patients, one was hospitalised with severe alcoholic hepatitis following MR assessment and so missed their planned outpatient biopsy and HVPG assessment. HVPG could not be computed from the available pressure measurements in the other five patients. One patient had non-cirrhotic portal hypertension and was included in the main analysis of spleen cT_1_ vs HVPG, but not in any of the analysis comparing HVPG to the other biomarkers. This patient was identified correctly by MR assessment as having non-cirrhotic portal hypertension (low liver cT_1_ of 767 ms; Ishak 1/6; high spleen cT_1_ of 1414 ms; HVPG 10 mmHg).

Transient elastography was performed in 18/19 patients, and results were reliable in 13 (76%). In those with unreliable results, the median/interquartile range ratio was unacceptably high (>0.3) in four patients and we failed to obtain 10 valid readings in one patient due to obesity, despite using the XL probe.

### Cohort characteristics

The majority of the patients were male (n = 15, 79%), and the main liver disease aetiologies were non-alcoholic fatty liver disease (n = 9, 47%), alcohol-related liver disease (n = 5; 26%) and viral hepatitis (n = 4, 21%). The median age was 57 and median BMI was 30.0 kg/m^2^ (Table 1). No patients were on non-selective beta-blockers or had comorbidities that affect the spleen (S1 Table). The median (IQR) HVPG was 9 mmHg (4–14). Ten patients were diagnosed with cirrhosis (Ishak 5–6). Liver biopsy samples had a median (IQR) length of 14 mm (9–22) and 7 (6–10) portal tracts. There were no adverse events related to the study procedures.

**Table 1:** Baseline characteristics and non-invasive scores in the presence or absence of clinically significant portal hypertension (HVPG ≥10 mmHg).

	All (n = 19)	HVPG <10 mmHg (n = 10)	HVPG ≥10 mmHg (n = 9)	P value
Age (years)	57 (47–68)	64 (50–74)	57 (45–63)	0.211
Male (%)	15 (79%)	9 (90%)	6 (67%)	0.213
BMI (kg/m^2^)	30.0 (24.2–32.3)	29.0 (23.9–33.4)	30.0 (24.6–32.2)	0.968
Cirrhosis (%)	10 (53%)	3 (30%)	7 (78%)	**0.037**
Ishak stage (0–6)	5 (1–6)	1 (1–2)	6 (5–6)	**0.028**
Aetiology				0.151*
NAFLD (%)	9 (47%)	4 (40%)	4 (44%)	
ASH (%)	5 (26%)	1 (10%)	3 (33%)	
Viral hepatitis (%)	4 (21%)	1 (10%)	2 (22%)	
Other (%)	2 (11%)	4 (40%)	2 (22%)	
Bilirubin (μmol/l)	12 (10–22)	12 (8–19)	13 (11–31)	0.447
ALT (IU/l)	44 (22–71)	42 (23–92)	49 (20–68)	0.604
ALP (IU/l)	188 (147–236)	175 (113–247)	205 (152–275)	0.447
Albumin (g/l)	41 (37–46)	43 (38–46)	40 (33–44)	0.315
AST (IU/l)	39 (32–76)	35 (26–70)	57 (37–78)	0.200
GGT (IU/l)	93 (40–277)	57 (25–226)	170 (60–279)	0.370
Platelets (x10^9^/l)	133 (112–216)	135 (121–242)	113 (98–202)	0.211
PT (s)	14.4 (13.1–16.6)	13.5 (12.1–15.1)	14.9 (14.4–17.3)	**0.035**
Liver stiffness (kPa)	21.3 (8.7–36.4)	11.9 (8.3–37.3)	35.1 (33.0–36.6)	0.148
AST/ALT ratio	1.09 (0.84–1.65)	0.88 (0.68–1.07)	1.39 (1.12–2.08)	**0.021**
APRI	0.72 (0.53–1.21	0.64 (0.52–0.75)	1.02 (0.71–1.57)	0.114
FIB-4	3.17 (1.84–4.01)	2.21 (1.37–3.51)	3.65 (2.41–5.46)	0.167
Child Pugh A	15 (79%)	8 (80%)	7 (78%)	ns
Child Pugh B	4 (21%)	2 (20%)	2 (22%)	
Child Pugh C	0	0	0	
Ascites present	2 (11%)	1 (10%)	1 (11%)	ns
**MR data**				
Spleen cT_1_ (ms)	1319 (1181–1414)	1212 (1169–1318)	1414 (1376–1448)	**0.001**
Liver cT_1_ (ms)	942 (867–1122)	874 (837–996)	1104 (938–1227)	**0.027**
Liver fat (%)	4.9 (1.3–13.7)	5.1 (1.3–13.4)	2.3 (1.4–15.4)	0.667

All data given as median (IQR) or frequency (%).

Abbreviations: HVPG, hepatic vein pressure gradient; BMI, body mass index; NAFLD, non-alcohol-related fatty liver disease; ASH, alcoholic steatohepatitis; ALT, alanine aminotransferase; ALP, alkaline phosphatase; AST, aspartate aminotransferase; GGT, gamma glutamyl transferase; PT, prothrombin time; APRI, AST to platelet ratio index; FIB-4, Fibrosis 4; ns, not significant

*Chi-squared test p value for the overall difference in liver disease aetiologies between groups

### Clinically significant portal hypertension

Eleven patients (57%) had portal hypertension (HVPG >5 mmHg) and nine (47%) had clinically significant portal hypertension (HVPG ≥10 mmHg). Patients with clinically significant portal hypertension had higher spleen cT_1_ (1414 vs 1212 ms; p = 0.001), liver cT_1_ (1104 vs 874 ms; p = 0.027), Ishak stage (6 vs 1; p = 0.028), prothrombin time (14.9 vs 13.5 s, p = 0.035) and AST/ALT ratio (1.39 vs 0.88; p = 0.021) than those without clinically significant portal hypertension (Table 1).

### Liver cirrhosis

Patients with cirrhosis had higher HVPG (12.8 vs 4.0 mmHg; p = 0.005) and spleen cT_1_ (1378 vs 1192 ms; p = 0.007) than those without cirrhosis (Table 2). Liver cT_1_ was numerically higher in patients with than without cirrhosis (1022 vs 876 ms; p = 0.146). No other non-invasive indices of fibrosis could differentiate between the two groups (Table 2).

**Table 2:** Non-invasive scores in the presence or absence of cirrhosis.

	All (n = 19)	Cirrhosis (n = 10)	No cirrhosis (n = 9)	P value
HVPG (mmHg)	9.0 (4.0–14.0)	12.8 (8.3–16.3)	4.0 (2.0–7.0)	**0.005**
Spleen cT_1_ (ms)	1319 (1181–1414)	1378 (1319–1443)	1192 (1166–1311)	**0.007**
Liver cT_1_ (ms)	942 (867–1122)	1022 (876–1202)	876 (829–1001)	0.146
Liver stiffness (kPa)	21.3 (8.7–36.4)	32.5 (12.0–35.8)	10.5 (8.3–58.7)	0.731
AST/ALT ratio	1.09 (0.84–1.65)	1.10 (0.82–1.99)	0.97 (0.82–1.44)	0.606
APRI	0.72 (0.53–1.21)	1.48 (0.89–2.08)	0.68 (0.39–1.031)	0.423
FIB-4	3.17 (1.84–4.01)	3.36 (1.94–5.06)	2.41 (1.70–3.64)	0.481

All data given as median (IQR). p-values for the comparison between patients with and without cirrhosis (Mann Whitney test).

Abbreviations: HVPG, hepatic vein pressure gradient; cT1, iron corrected T1, AST, aspartate aminotransferase; ALT, alanine aminotransferase; APRI, AST to platelet ratio index; FIB-4, Fibrosis 4.

### Associations with HVPG

**Fig 3:**
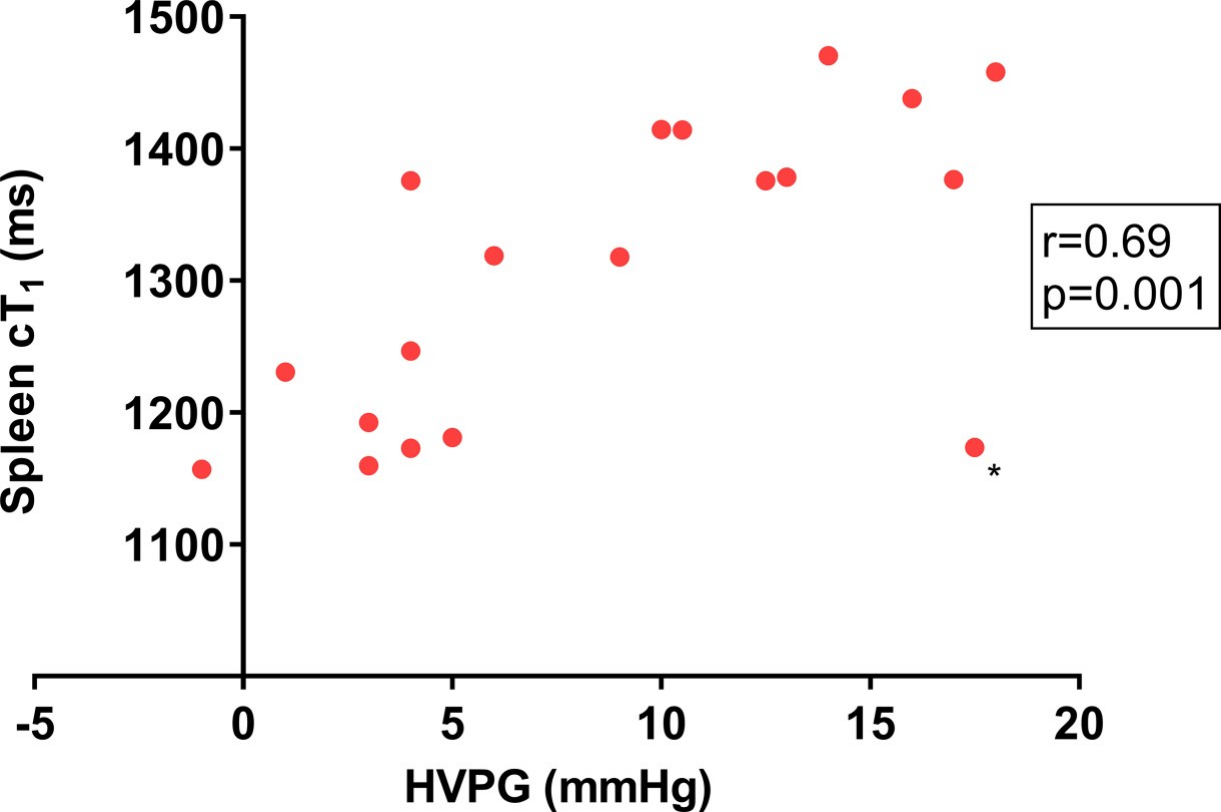
Spleen iron corrected T_1_ (cT_1_) correlation with the hepatic vein pressure gradient. There was a strong association between spleen cT_1_ and portal pressure as measured by the hepatic vein pressure gradient (HVPG, r = 0.69; p = 0.001). *Data from an outlying patient whose HVPG measurement was out of keeping with the rest of the clinical data raising the possibility of an inaccurate HVPG measurement.

Spleen cT_1_ was the only non-invasive biomarker tested to have a significant correlation with HVPG (r = 0.69; p = 0.001) (Fig 3, Table 3). There was a trend in the association of liver cT1 and HVPG (r = 0.40; p = 0.101; S1 Fig).

**Table 3:** Non-invasive scores and their correlation with HVPG.

	Median value (IQR) n = 19	Correlation with HVPG (r)	P value
Spleen cT_1_ (ms)	1319 (1181–1414)	0.69	**0.001**
Liver cT_1_ (ms)	942 (867–1122)	0.40	0.105
Liver stiffness (kPa)	21.3 (8.7–36.4)	0.35	0.244
PT (s)	14.4 (13.1–16.6)	0.36	0.127
AST/ALT ratio	1.09 (0.84–1.65)	0.47	0.058
APRI	0.72 (0.53–1.21)	0.26	0.309
FIB-4	3.17 (1.84–4.01)	0.27	0.295

Abbreviations: HVPG, hepatic vein pressure gradient; cT_1_, iron corrected T_1_, PT, prothrombin time; AST, aspartate aminotransferase; ALT, alanine aminotransferase; APRI, AST to platelet ratio index; FIB-4, Fibrosis 4.

### Linear regression

Spleen cT_1_ (p = 0.002), liver cT_1_ (p = 0.065), Ishak stage (p = 0.005) and AST/ALT ratio (p = 0.058) were all predictors of HVPG with p<0.1 on univariate analysis (S2 Table). Of the non-invasive variables, only spleen cT_1_ was identified as an independent predictor of HVPG (p = 0.03).

### Logistic regression

Spleen cT_1_ (p = 0.014), liver cT_1_ (p = 0.032), Ishak stage (p = 0.038) and AST/ALT ratio (p = 0.064) and prothrombin time (p = 0.065) were all predictors of clinically significant portal hypertension with p<0.1. On multivariate analysis, spleen cT_1_ (p = 0.069) was the only parameter to improve the model with p<0.1. (S3 Table).

### Diagnostic accuracy for portal hypertension

**Fig 4:**
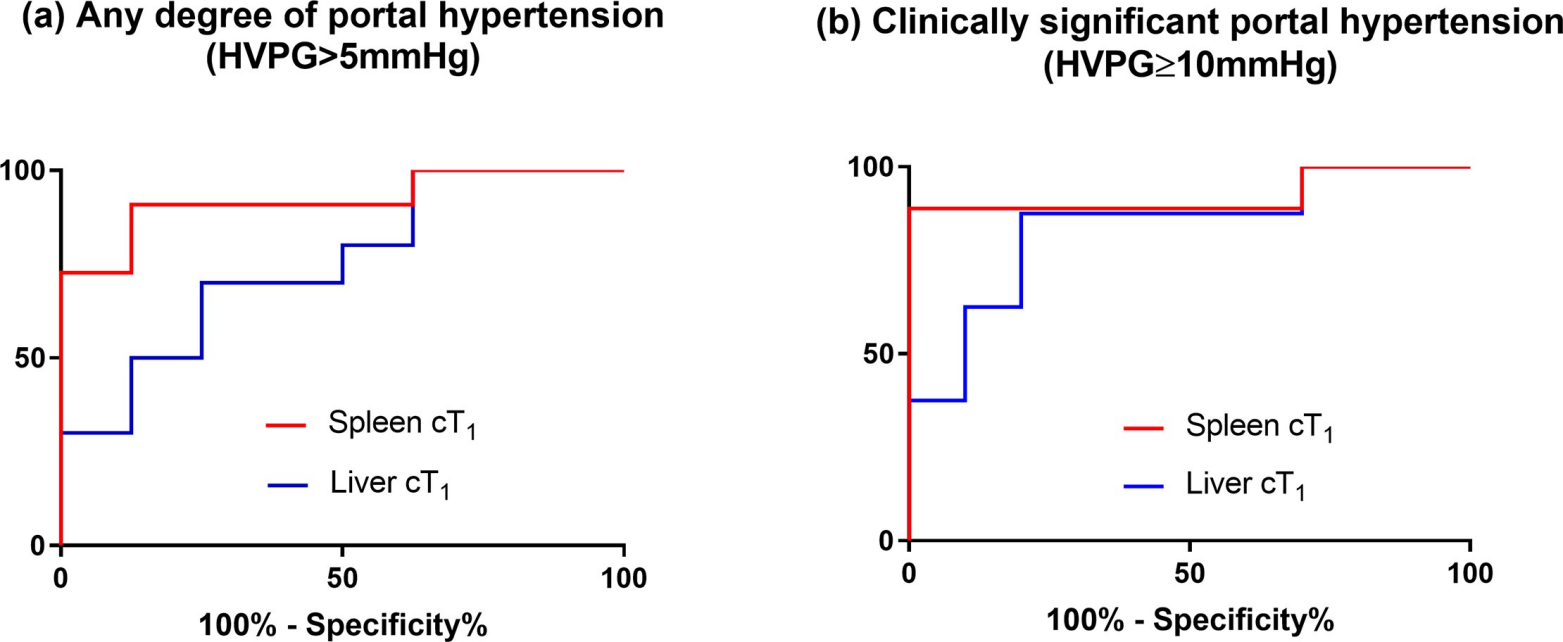
Diagnostic accuracy of spleen and liver iron corrected T_1_ (cT_1_) for portal hypertension severity assessment. (A) Receiver operating curves (ROC) of spleen and liver cT_1_ for the diagnosis of any degree of portal hypertension. There was a significant diagnostic accuracy for spleen cT_1_ with an area under the ROC (AUROC) of 0.92 (p = 0.002). Liver cT_1_ had an AUROC of 0.73 with a trend towards significance (p = 0.110).(B) ROC curves of spleen and liver cT1 for the diagnosis of clinically significant portal hypertension. Both variables had significant diagnostic accuracy with AUROC of 0.92 (p = 0.002) for spleen cT1 and 0.81 (p = 0.026) for liver cT1.

Spleen cT_1_ had excellent accuracy for the diagnosis of any degree of portal hypertension and clinically significant portal hypertension with area under the receiver operating curve (AUROC) 0.92 (p = 0.002) for both (Fig 4, Table 4). Liver cT_1_ showed significant accuracy for the diagnosis of clinically significant portal hypertension with AUROC 0.81 (p = 0.026). Liver stiffness did not yield statistically significant results (p = 0.123). For the diagnosis of clinically significant portal hypertension, a spleen cT_1_ threshold of 1376 ms had 89% sensitivity, 100% specificity, 100% positive predictive value (PPV) and 91% negative predictive value (NPV), and a liver cT_1_ threshold of 909 ms had 88% sensitivity and 70% specificity, 73% PPV and 88% NPV (Table 4).

**Table 4:** Accuracy of spleen and liver cT1 for the diagnosis of HVPG severity categories.

	AUROC (95%CI)	P value	Cut-off	Sensitivity (%)	Specificity (%)	PPV (%)	NPV (%)
**Spleen cT**_**1**_							
HVPG >5 mmHg	0.92 (0.79–1.00)	0.002	1282 ms	91	88	91	88
HVPG ≥10 mmHg	0.92 (0.77–1.00)	0.002	1376 ms	89	100	100	91
**Liver cT**_**1**_							
HVPG >5 mmHg	0.73 (0.49–0.96)	0.110	909 ms	70	63	70	63
HVPG ≥10 mmHg	0.81 (0.61–1.00)	0.026	909 ms	88	70	73	88
**Liver stiffness**							
HVPG >5 mmHg	0.71 (0.38–1.00)	0.668	12.0 kPa	100	71	75	100
HVPG ≥10 mmHg	0.78 (0.51–1.00)	0.123	26.9 kPa	100	78	67	100

Abbreviations: AUROC, area under the receiver operating characteristic curve; 95% CI, 95% confidence interval; PPV, positive predictive value; NPV, negative predictive value; HVPG, hepatic vein pressure gradient

### Outlying data

There was one outlying dataset marked with an asterisk on Fig 3. The results from this patient were included in the analysis, however, their HVPG measurement of 17.5 mmHg was out of keeping with the rest of their clinical data. Cirrhosis was suspected based on a high liver stiffness of 23.6 kPa (IQR 5.4, success 91%), however, measurement as part of the study was low and unreliable (median/IQR >0.3; median 4.1 kPa; IQR 1.6). The biopsy showed only mild fibrosis (Ishak 2) and there were no radiological features of cirrhosis or portal hypertension. Furthermore, liver synthetic function (albumin 40 g/l, prothrombin time 14.4 s) and platelet count (216 x10^9^/l) were normal. It is possible that the HVPG measurement in this case was inaccurate. Exclusion of this data point from the analysis would have improved diagnostic accuracy for clinically significant portal hypertension for spleen cT_1_ (r = 0.87, p<0.001; AUROC 1, p<0.001) and liver cT_1_ (r = 0.54, p = 0.027; AUROC 0.89, p = 0.008).

## Discussion

This is the first study to describe spleen cT_1_ as a non-invasive and accurate biomarker of portal hypertension. Even though this was a small proof of principle study, spleen cT_1_ was found to have a strong correlation with HVPG (r = 0.69, p = 0.001), be an independent predictor of HVPG and have a high diagnostic accuracy for any degree of portal hypertension and clinically significant portal hypertension (AUROC 0.92 for both). A spleen cT_1_ threshold of 1376 ms at 3T was able to diagnose clinically significant portal hypertension with 89% sensitivity and 100% specificity. Furthermore, spleen cT_1_ performed better than other non-invasive markers both for the assessment of portal hypertension and for the diagnosis of cirrhosis.

Portal hypertension develops due to increased hepatic resistance and portal flow. The physical effects of liver fibrosis and dynamic effects of increased vascular tone within the hepatic venous sinusoids cause hepatic resistance, whilst angiogenesis and systemic vasodilatation increase portal blood flow[27].

The performance of spleen cT_1_ was superior to that of liver biomarkers (liver cT_1,_ liver stiffness, AST/ALT ratio, APRI and FIB-4) probably because liver fibrosis reflects just one element of portal hypertension pathophysiology, whereas spleen cT_1_ directly reflects the consequence of portal hypertension as a whole.

Spleen parameters are used routinely to assess for portal hypertension. Clinical examination and imaging can assess for splenomegaly, but both these approaches are subjective and hence lack sensitivity[28]. Thrombocytopaenia resulting from portal hypertension-related hypersplenism is also assessed routinely in clinical practice and maybe useful particularly when incorporated into prediction indices with other non-invasive tests[15, 25]. In this study however, platelet count was not predictive of HVPG in univariate analysis and did not differentiate patients with and without clinically significant portal hypertension (113x10^9^/l vs 135x10^9^/l, p = 0.21).

Spleen stiffness has also been assessed alongside liver stiffness as a biomarker of portal hypertension, with greater accuracy in viral hepatitis than other liver disease aetiologies. In a seminal study using transient elastography in patients with chronic hepatitis C, HVPG was closely associated with spleen stiffness (R^2^ = 0.78, p<0.001) and liver stiffness (R^2^ = 0.70, p<0.001)[29]. In another large study where 76% of the patients had viral hepatitis, liver stiffness had an AUROC of 0.88 for the diagnosis of clinically significant portal hypertension[15]. Applications of these techniques to mixed patient cohorts, however, have produced less impressive results. In one study, where only 30% of the patients had viral hepatitis, spleen stiffness had only a modest association with HVPG (r = 0.433, p = 0.001) while liver stiffness did not correlate at all (r = 0.178, p = 0.2)[30].

Shear wave elastography is a technique that could also be useful in patients with ascites and obesity. The equipment to perform shear wave elastography is usually incorporated into standard ultrasound machines. Unlike transient elastography, the measurements are taken at the time of ultrasound examinations for anatomical assessment and therefore require a separate appointment. The diagnostic accuracy for clinically significant portal hypertension was modest for liver and spleen stiffness with respective AUROC of 0.79 and 0.72 using shear wave elastography, compared with AUROC of 0.78 and 0.63 using transient elastography[31]. At meta-analysis, shear wave elastography was found to have good accuracy for the diagnosis of CSPH with an AUROC of 0.88[32].

MR elastography (MRE) has also been used to study spleen and liver stiffness for the assessment of portal hypertension, with excellent pre-clinical results[17, 33]. In a patient study, however, MRE resulted only in modest association between HVPG and spleen (r = 0.53, p = 0.004) and liver (r = 0.44, p = 0.05) visco-elastic parameters[34]. Another MR study examined multiple parameters including liver and spleen T_1_, splanchnic and portal vein flow measurements and described a linear regression model including liver T_1_ and splenic artery velocity that correlated closely with HVPG[35]. The long data acquisition with this protocol (1 hour), however, limits its clinical applicability.

The results from our study compare favourably with these other markers, particularly considering the mixed aetiologies in our patient cohort. In addition, unlike MRE, our multi-parametric MR techniques do not require additional hardware and unlike transient elastography, our measures can be obtained independent of body habitus. Furthermore, our multi-parametric MR protocol is completely non-invasive, highly reproducible and scans can be performed and analysed quickly [19].

The current Baveno consensus guidelines[36] recommend that diagnosis of compensated advanced chronic liver disease (cACLD) can be made based on liver stiffness measurements by transient elastography. Invasive tests can be used where there is diagnostic doubt or for confirmation. Liver stiffness and platelet cut-offs are also recommended for diagnosis of CSPH in patients with viral aetiologies and for safely avoiding screening endoscopy for oesophageal varices. In patients with cACLD of all aetologies, imaging showing presence of collateral vessels is sufficient to diagnose CSPH, which is particularly relevant to our imaging biomarkers that could complement anatomical assessment.

We envision portal hypertension assessment by spleen cT_1_ to complement the existing abilities of multi-parametric MR to quantify liver inflammation, fibrosis, fat and iron [19] and risk stratify for clinical outcomes [21]. Such multifactorial assessment was demonstrated in one patient with non-cirrhotic portal hypertension, where liver cT_1_ (767 ms) was able to rule out cirrhosis, and spleen cT_1_ (1414 ms) detected clinically significant portal hypertension correctly. The ability to assess multiple parameters in one simple test makes this technology very attractive to explore further. In clinical practice, the information provided by this technology may reduce the need for liver biopsies and HVPG measurements, which are invasive and costly (see S4 Table for comparison of MR features with alternative tests). In the context of clinical trials, this technology may help to accelerate drug development as spleen cT_1_ correlated strongly with HVPG, an FDA recognised end-point. Recruitment and retention of participants to trials may improve, as utilisation of this technology would avoid the need for invasive procedures like biopsy and HVPG measurement, which are unattractive to patients.

### Study limitations

The small sample size limits the interpretation of the study as proof of concept only. Unlike most HVPG studies, patients in our cohort were recruited based on the suspicion of cirrhosis, which was confirmed in 53%. Consequently, 43% of patients had no portal hypertension and the highest HVPG was 18 mmHg. Larger studies specifically in cirrhotic patients are needed, ideally including HVPGs up to 40 mmHg [37]. Furthermore, measurement of T_1_ is dependent on magnet field strength and the specific method used for its estimation. LiverMultiScan allows standardisation across magnet vendors and field strengths, which will have to be tested in larger multi-site studies.

The accuracy of cT_1_ measurements to reflect water content could be biased by other physiological parameters [38], but when applied to the spleen this parameter was the only independent predictor of HVPG. Furthermore, the iron correction algorithm used here was developed for correction of liver data, without any further modifications to reflect spleen physiology. Closer examination of factors that can affect T_1_ measurement in the liver and spleen and the creation of organ specific correction mechanisms should be the focus of further studies.

### Conclusions

This study showed that spleen cT_1_ correlates strongly with HVPG and therefore, could be a useful biomarker of portal hypertension. Formal recommendations about clinical application should be made based on larger studies to explore the relationship of spleen cT_1_, splenic volume and other MR biomarkers with a wider range of portal pressures. Future studies should also focus on the ability of spleen cT_1_ to predict the presence of oesophageal varices and other clinical outcomes.

## Supporting information

S1 Checklist STARD checklist. (DOCX)

S1 Dataset (XLSX)

S1 Text Details of recruitment strategy. (DOCX)

S1 Fig Liver iron corrected T1 correlation with the hepatic vein pressure gradient. (PDF)

S1 Table Extrahepatic comorbidities and medications of patients included in the final analysis. (DOCX)

S2 Table Linear regression analyses for the prediction of hepatic vein pressure gradient. (DOCX)

S3 Table Logistic regression analysis for the diagnosis of clinically significant portal hypertension. (DOCX)

S4 Table Comparison of MR features with alternative approaches. (DOCX)
