# Supplementary material for: Non-invasive assessment of portal hypertension by multi-parametric magnetic resonance imaging of the spleen: A proof of concept study
Source: PLoS One. 2019 Aug 20;14(8):e0221066. doi: 10.1371/journal.pone.0221066 (PMC6701782; doi:10.1371/journal.pone.0221066)
Supplement: S1 Text — (DOCX) [file pone.0221066.s003.docx]

**S2 Text. Details of recruitment strategy**

Patients were recruited from the hepatology service at the John Radcliffe hospital. Patients who were referred for liver biopsy for suspected cirrhosis were asked by their clinician whether they would be happy to receive information about the study and those who agreed were sent a patient information leaflet. Patients who agreed to take part and met the inclusion and exclusion criteria were recruited. Data were only collected once the patients signed the consent form for the study in line with good clinical practice. Data were not collected on how many patients were approached by the clinicians, how many of these agreed to receive information about the study and how many of those that received information did not agree to agree to take part.
