# Supplementary material for: Non-invasive assessment of portal hypertension by multi-parametric magnetic resonance imaging of the spleen: A proof of concept study
Source: PLoS One. 2019 Aug 20;14(8):e0221066. doi: 10.1371/journal.pone.0221066 (PMC6701782; doi:10.1371/journal.pone.0221066)
Supplement: S1 Fig — (PDF) [file pone.0221066.s004.pdf]

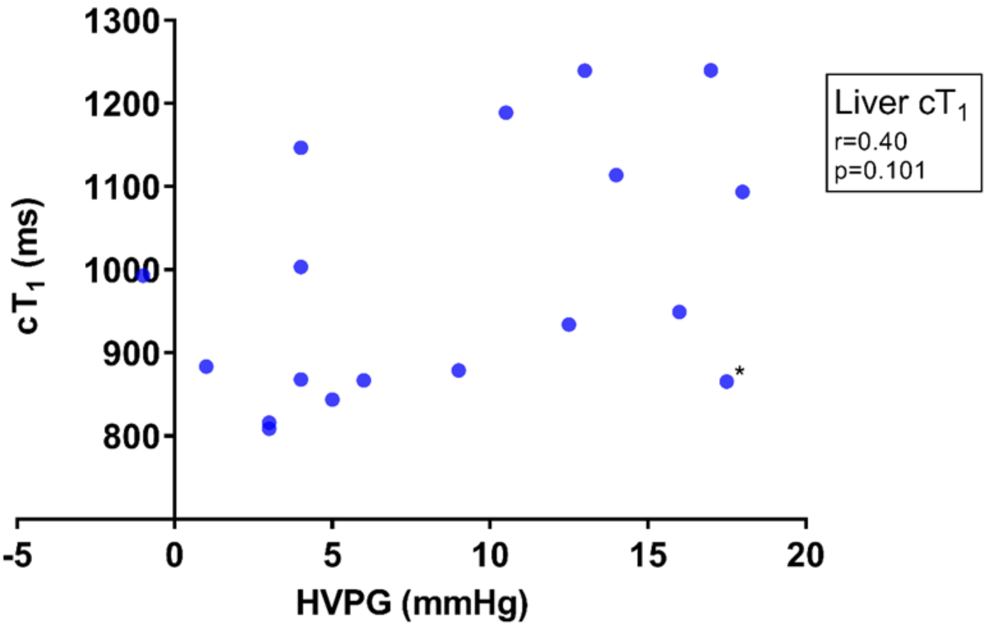

Figure. Liver iron corrected T_1 correlation with the hepatic vein pressure gradient.

There was a trend towards an association between liver cT_1 and HVPG (r=0.40; p=0.105).

*Data from an outlying patient whose HVPG measurement was out of keeping with the rest of the clinical data raising the possibility of an inaccurate HVPG measurement.
