# Supplementary material for: Non-invasive assessment of portal hypertension by multi-parametric magnetic resonance imaging of the spleen: A proof of concept study
Source: PLoS One. 2019 Aug 20;14(8):e0221066. doi: 10.1371/journal.pone.0221066 (PMC6701782; doi:10.1371/journal.pone.0221066)
Supplement: S1 Table — (DOCX) [file pone.0221066.s005.docx]

| **S3 Table:** Extrahepatic comorbidities and medications of patients included in the final analysis | | |
| --- | --- | --- |
| **id** | **Extrahepatic comorbidity** | **Medications** |
| 4.1 | Type 2 diabetes mellitus, hypothyroidism | Omeprazole, Thyroxine, Metformin, Ferrous fumarate, Tramadol. |
| 4.4 | Hypertension, benign prostatic hypertrophy, tinnitus, anxiety | Doxazosin, Tamsulosin |
| 4.5 | Vitamin D deficiency | Ursodeoxecholic acid, fenofibrate, colecalciferol |
| 4.6 | Coronary artery disease, coronary stents, benign prostatic hypertrophy | Aspirin, simvastatin, finasteride, bendroflumethiazide, omeprazole, GTN spray as needed, naproxen as needed |
| 4.7 | Anxiety, depression | Methadone, vitamin B complex strong, thiamine, haloperidol, omeprazole |
| 4.8 | Psoriasis | Dovobet ointment, tacrolimus ointment |
| 4.9 | Crohn’s disease, hypertension, atrial fibrillation | Bisoprolol, digoxin, ramipril, ferrous sulphate, lansoprazole, atorvastatin, methotrexate once a week, folic acid once a week, spironolactone, ciprofloxacin. |
| 4.10 | Previous renal transplant (IgA nephropathy), hypertension, angina | Furosemide, doxazosin, aranesp, losartan, flecainide, aspirin, isosorbide mononitrate, atenolol, allopurinol, ciclosporin, prednisolone, calcichew, alfacalcidol |
| 4.11 | Hypertension, chronic obstructive pulmonary disease, hypercholesterolaemia | Hormone replacement therapy, omeprazole, atorvastatin, felodipine, citalopram, inhalers |
| 4.12 | Hypertension, previous renal artery stent | Simvastatin, ramipril, citalopram, clopidogrel |
| 4.13 | Hypertension, diabetes mellitus, prior endometriosis, hysterectomy | Atenolol, gabapentin, lansoprazole, sertraline, hormone replacement therapy, metformin, amitriptyline. |
| 4.14 | Pulmonary sarcoidosis, pulmonary embolism, hypertension, benign prostatic hypertrophy | Prednisolone, warfarin, tamsulosin, ramipril, simvastatin, inhalers, lansoprazole, fluoxetine, oxybutynin. |
| 4.16 | Hypertension, obstructive sleep apnoea, ulcerative colitis | Ramipril, citalopram, prednisolone |
| 4.17 | Type 2 diabetes mellitus, hypertension | Metformin, gliclazide, Lisinopril, doxazosin |
| 4.19 | Ulcerative colitis | Pentasa, azathioprine |
| 4.20 | Morbid obesity, Crohn’s disease, previous right hemicolectomy | Adalimumab , tramadol, amitriptyline |
| 4.21 | Type 2 diabetes mellitus, hypertension, hypercholesterolaemia | Metformin, atorvastatin, perindopril |
| 4.22 | Hypertension, dyslipidaemia, cerebrovascular disease | Rosuvastatin, nifedipine LA, clopidogrel, aspirin. |
| 4.23 | Nil | Nil |
