# Supplementary material for: Non-invasive assessment of portal hypertension by multi-parametric magnetic resonance imaging of the spleen: A proof of concept study
Source: PLoS One. 2019 Aug 20;14(8):e0221066. doi: 10.1371/journal.pone.0221066 (PMC6701782; doi:10.1371/journal.pone.0221066)
Supplement: S2 Table — (DOCX) [file pone.0221066.s006.docx]

| **S5 Table:** Linear regression analyses for the prediction of hepatic vein pressure gradient | | | |
| --- | --- | --- | --- |
|  | **Univariate** | | **Multivariate** |
| **Variable** | **R^2^** | **p** | **p** |
| **Spleen cT**_1_ **(ms)** | **0.44** | **0.002** | **0.03** |
| **Liver cT_1_ (ms)** | **0.2** | **0.065** | **0.57** |
| Liver fat (%) | 0.005 | 0.769 |  |
| **Ishak stage (0-6)** | **0.37** | **0.005** | ***** |
| Liver stiffness measurement (kPa) | 0.023 | 0.62 |  |
| Age (yrs) | 0.01 | 0.69 |  |
| Body mass index (Kg/m^2^) | 0.014 | 0.64 |  |
| Bilirubin | 0.021 | 0.56 |  |
| ALT (iu/l) | 0.016 | 0.61 |  |
| Alkaline phosphatase (iu/l) | 0.006 | 0.75 |  |
| Albumin (g/l) | 0.033 | 0.45 |  |
| Gamma glutamyl transferase (iu/l) | 0.004 | 0.81 |  |
| AST (iu/l) | 0.00 | 0.96 |  |
| Platelet count (x10^9^/l) | 0.029 | 0.49 |  |
| Prothrombin time (s) | 0.132 | 0.13 |  |
| **AST/ALT ratio** | **0.219** | **0.058** | **0.21** |
| AST to platelet ratio index (APRI) | 0.01 | 0.89 |  |
| Fibrosis 4 (FIB-4) | 0.034 | 0.48 |  |
| Child Pugh score (5-15) | 0.012 | 0.66 |  |
| Parameters with p<0.1 in bold.  **Abbreviations:** cT_1_, iron corrected T_1_, AST, aspartate aminotransferase; ALT, alanine aminotransferase.  * Only non-invasive variables were included in the multivariate model, therefore Ishak was excluded. | | | |
