# Supplementary material for: Non-invasive assessment of portal hypertension by multi-parametric magnetic resonance imaging of the spleen: A proof of concept study
Source: PLoS One. 2019 Aug 20;14(8):e0221066. doi: 10.1371/journal.pone.0221066 (PMC6701782; doi:10.1371/journal.pone.0221066)
Supplement: S3 Table — (DOCX) [file pone.0221066.s007.docx]

| **S6 Table:** Logistic regression analysis for the diagnosis of clinically significant portal hypertension | | |
| --- | --- | --- |
|  | **Univariate** | **Multivariate** |
| **Variable** | **p** | **p** |
| **Spleen cT_1_ (ms)** | **0.014** | **0.069** |
| **Liver cT_1_ (ms)** | **0.032** | **0.640** |
| Liver fat (%) | 0.619 |  |
| **Ishak stage (0-6)** | **0.038** | ***** |
| Liver stiffness measurement (kPa) | 0.815 |  |
| Age (yrs) | 0.396 |  |
| Body mass index (Kg/m^2^) | 0.570 |  |
| Bilirubin | 0.481 |  |
| Alanine aminotransferase (iu/l) | 0.609 |  |
| Alkaline phosphatase (iu/l) | 0.709 |  |
| Albumin (g/l) | 0.254 |  |
| Gamma glutamyl transferase (iu/l) | 0.947 |  |
| Aspartate aminotransferase (iu/l) | 0.903 |  |
| Platelet count (x10^9^/l) | 0.302 |  |
| **Prothrombin time (s)** | **0.065** | **0.228** |
| **AST/ALT ratio** | **0.064** | **0.237** |
| AST to platelet ratio index (APRI) | 0.767 |  |
| Fibrosis 4 (FIB-4) | 0.480 |  |
| Child Pugh score (5-15) | 0.597 |  |
| Parameters with p<0.1 in bold  **Abbreviations:** cT_1_, iron corrected T_1_, AST, aspartate aminotransferase; ALT, alanine aminotransferase.  *Only non-invasive variables were included in the multivariate model, therefore Ishak was excluded. | | |
