# Supplementary material for: Non-invasive assessment of portal hypertension by multi-parametric magnetic resonance imaging of the spleen: A proof of concept study
Source: PLoS One. 2019 Aug 20;14(8):e0221066. doi: 10.1371/journal.pone.0221066 (PMC6701782; doi:10.1371/journal.pone.0221066)
Supplement: S4 Table — (DOCX) [file pone.0221066.s008.docx]

| **S7 Table:** Comparison of MR features with alternative approaches | | | |
| --- | --- | --- | --- |
|  | **MRI** | **Elastography** | **Invasive test (HVPG)** |
| **Cost** | Medium* | Medium* | High |
| **Availability** | High  *MRI available in most hospitals* | High  *TE available in most hospitals* | Low / Medium  *Only in specialist centres* |
| **Invasiveness** | Low | Low | High |
| **Duration of test** | 0-15 minutes | 0-15 minutes | Day case procedure  *6-8 hours including recovery time* |
| **Expertise needed** | Medium  *radiographer* | Low / Medium  *Trained operator* | High  *Interventional radiologist* |
| **Applicability** | High  *Contra-indicated in patients with pacemakers / implanted devices* | Medium / High  *Not successful in all patients (ascites, obesity)* | High  *Can be performed in most patients* |
| **Waiting time for test** | Variable  *Patients have to attend dedicated visit for MR scan* | Variable  *Usually done at the time of clinic appointment, so waiting time for test is dependent on clinic waiting list* | Long  *Depends on availability of specialist* |
| *The costs will vary between countries and types of health care systems. In the context of the UK where this study was conducted, the cost of an MRI of one area without contrast is £141 and the cost of ultrasound elastography is £199 (https://improvement.nhs.uk/resources/reference-costs/#rc1718). | | | |
